# Red Cell Distribution Width-to-Platelet Count Ratio as an Early Marker of Severity in Acute Pancreatitis: A Diagnostic Accuracy Study at a Tertiary Care Center

**DOI:** 10.7759/cureus.116212

**Published:** 2026-09-14

**Authors:** Sumedha Ghosh, Ramchandra Besra, Pankaj Bodra, Swetav Suman, Mrinank Harshvardhan, Anmol Kujur, Prem P Choudhary, Dipanjan Saha, Namrata Dutta, Arunav Roy

**Affiliations:** 1 General Surgery, Rajendra Institute of Medical Sciences, Ranchi, IND; 2 Cardiology, Fortis Healthcare, Kolkata, IND; 3 Plastic Surgery, Rajendra Institute of Medical Sciences, Ranchi, IND

**Keywords:** acute pancreatitis, biomarkers, platelet count, prognosis, red cell distribution width, severity of illness index

## Abstract

Introduction: Early identification of clinically significant acute pancreatitis is essential for timely escalation of care, yet established scoring systems are multiparametric, and several require 48-hour data. The red cell distribution width (RDW)-to-platelet count ratio is a composite index derived from the routine complete blood count that may reflect the systemic inflammatory burden of the disease. This study assessed its accuracy for identifying moderately severe or severe disease at admission.

Methods: Consecutive adults admitted with acute pancreatitis were enrolled on accrual at a tertiary care center. The ratio was calculated from the admission RDW and platelet count. Disease severity was classified using the Revised Atlanta Classification; the target condition for the accuracy analysis was moderately severe or severe disease versus mild disease. Associations with the Ranson score, intensive care admission, organ failure, and length of hospital stay were examined, and discrimination was assessed using the area under the receiver operating characteristic curve.

Results: Among 142 patients (mean age: 43 years; 86 (60.6%) men), 47 (33.1%) patients had moderately severe or severe disease. The ratio rose progressively across severity categories and was higher in patients requiring intensive care and those with organ failure (all p < 0.001). At a cut-off of 0.0601, it identified moderately severe or severe disease with sensitivity of 45/47 (95.7%), specificity of 74/95 (77.9%), and negative predictive value of 74/76 (97.4%) (area under the curve: 0.931).

Conclusions: The RDW-to-platelet ratio is a simple, inexpensive admission marker that showed high sensitivity and negative predictive value for clinically significant severity and may support early risk stratification, pending external validation alongside established assessment.

## Introduction

Acute pancreatitis is a common gastrointestinal emergency with a clinical course ranging from mild, self-limiting inflammation to severe disease complicated by persistent organ failure and death [1]. Its incidence has risen over recent decades, imposing a substantial burden on health systems, particularly where intensive care capacity is limited [2,3]. The Revised Atlanta Classification stratifies the disease into mild, moderately severe, and severe categories according to the presence and duration of organ failure and local or systemic complications [4]. Although most patients follow a mild course, a clinically important minority progress to moderately severe or severe disease, in whom prolonged admission, intensive care, and mortality are concentrated [5]. Early risk stratification is therefore central to management.

Several scoring systems predict severity, including the Ranson criteria, the Acute Physiology and Chronic Health Evaluation (APACHE), the Glasgow score, and the Bedside Index for Severity in Acute Pancreatitis (BISAP) [6-8]. In practice, these tools are limited: many require multiple parameters, and some depend on 48-hour data that delay definitive stratification during the critical early hours [7]. A meta-analysis has confirmed the modest early discrimination of composite scores, and imaging-based indices are not always immediately available [9,10]. Consequently, there is continued interest in simple, inexpensive markers obtainable at admission.

Inflammation-based indices derived from the complete blood count are attractive for this purpose because they are universally available and require no additional cost. Red cell distribution width (RDW), a measure of variability in erythrocyte size, is associated with systemic inflammation and impaired erythropoiesis and correlates with inflammatory biomarkers [11]. It has shown prognostic value for severity, mortality, and persistent organ failure in acute pancreatitis [12-15]. Platelets participate in inflammation beyond hemostasis, and their consumption during severe inflammation may lower the platelet count. The RDW-to-platelet ratio combines these two changes into a single index; because inflammation tends to raise the numerator while lowering the denominator, the ratio may amplify the discriminatory signal of either component alone. Preliminary work has reported prognostic value for the ratio in acute pancreatitis [16-18], and related RDW ratios using serum calcium or albumin have shown comparable performance [19-21]. The ratio was first described as a predictor of hepatic fibrosis and cirrhosis in chronic hepatitis B [22] and has since been applied to inflammatory disease more broadly.

Despite this interest, prospective data on the ratio in acute pancreatitis remain limited, particularly from the Indian subcontinent, and reported cut-off values vary widely, partly because studies express the platelet denominator in different units, rendering absolute cut-offs not comparable. Robust prospective evaluation against a standardized severity framework is therefore needed. The primary objective was to assess the accuracy of the admission RDW-to-platelet ratio for identifying moderately severe or severe acute pancreatitis. Secondary objectives were to examine its relationship with the Ranson score and with intensive care admission, organ failure, and length of hospital stay.

## Materials and methods

Study design and setting

Consecutive adults admitted with acute pancreatitis to the Department of General Surgery of a tertiary care teaching hospital were enrolled on accrual over 18 months, following approval of the protocol by the Institutional Ethics Committee. The study was reported in accordance with the Standards for Reporting of Diagnostic Accuracy Studies (STARD). Recruitment ran from June 2025 to March 2026.

Participants

Patients older than 18 years, of either sex, diagnosed with acute pancreatitis and admitted within 48 hours of symptom onset were eligible. Patients with a known or clinically apparent hematological or coagulation disorder, acute-on-chronic pancreatitis, advanced malignancy or ongoing chemoradiotherapy, and pregnant women were excluded (Table 1).

**Table 1:** Inclusion and exclusion criteria Criteria were applied at the time of screening.

Category	Criterion
Inclusion	Age older than 18 years
	Either sex
	Diagnosis of acute pancreatitis
	Admission within 48 hours of symptom onset
Exclusion	Known or clinically apparent hematological or coagulation disorder
	Acute-on-chronic pancreatitis
	Advanced malignancy or ongoing chemoradiotherapy
	Pregnancy

Index test

The RDW-to-platelet ratio was calculated as the RDW (%) divided by the platelet count (× 10⁹/L). Venous samples drawn at admission, 24 hours, and 48 hours were analyzed on the laboratory’s automated hematology analyzer (HORIBA Yumizen H2500; HORIBA Ltd., Kyoto, Japan); the admission value was the primary index test. The platelet unit used here (× 10⁹/L) is stated explicitly because cut-off values are not comparable across studies that express the denominator as an absolute count.

Reference standard and target condition

Disease severity was classified using the Revised Atlanta Classification into mild, moderately severe, and severe disease, using clinical, biochemical, and imaging criteria, with the contrast-enhanced computed tomography (CECT) severity index assessed after 72 hours [4,23]. Organ failure was defined using the modified Marshall score (≥2 in the respiratory, cardiovascular, or renal system), transient if resolving within 48 hours and persistent if exceeding 48 hours; local complications comprised pancreatic or peripancreatic necrosis and fluid collections. Severity was assigned once the complete admission course was known, so that the 48-hour persistence criterion could be applied. Because the Atlanta framework is three-tiered but the accuracy analysis requires a binary target, the target condition was defined a priori as moderately severe or severe disease (i.e., any clinically significant severity beyond mild) versus mild disease. The Ranson score was calculated from 48-hour data as a comparator [24]. The index test and the reference standard were assessed independently: the RDW-to-platelet ratio was not disclosed to the clinicians who assigned the Atlanta severity category, and severity classification was based on clinical, biochemical, and imaging criteria without reference to the ratio. Because Atlanta severity depends principally on the presence and duration of organ failure and on local or systemic complications, the 13 patients who did not undergo CECT were classified from clinical, biochemical, and organ failure criteria, with CECT contributing to the identification of local complications where performed. Although early identification of severity is prognostic in nature, diagnostic accuracy methodology (sensitivity, specificity, and the area under the receiver operating characteristic curve) was applied to quantify how well the admission ratio classified this target condition.

Sample size

Using the method for diagnostic tests that incorporates sensitivity and specificity [25], with an assumed sensitivity of 80%, specificity of 70%, a 95% confidence level, and a 10% margin of error, the required sample size was 142. As the target condition was present in a minority of patients, precision for the sensitivity estimate rests on this subgroup and is reflected in the confidence intervals reported below.

Data collection

Demographic, clinical, laboratory, radiological, and severity data were recorded on a pre-tested proforma. The ratio was derived at admission, 24 hours, and 48 hours; Ranson scores used 48-hour data.

Statistical analysis

Analyses used jamovi (version 2.6.44; The jamovi project, Sydney, Australia). The distribution of continuous variables was assessed using the Shapiro-Wilk test; skewed variables were summarized as median (interquartile range (IQR)) and compared using the Mann-Whitney U or Kruskal-Wallis test, with Dwass-Steel-Critchlow-Fligner pairwise comparisons following a significant Kruskal-Wallis result, and approximately symmetric variables as mean (standard deviation (SD)). Categorical variables were compared using the Pearson chi-square test, with degrees of freedom and Cramér’s V reported alongside each test statistic; the minimum expected cell count was 6.6, so no exact test was required. Correlations used the Spearman coefficient. Diagnostic performance was assessed using the area under the receiver operating characteristic curve, with an optimal cut-off identified by the Youden index; sensitivity, specificity, and predictive values were computed at this cut-off. The 95% confidence intervals (CIs) were derived using the Wilson method for proportions and bootstrap resampling for the area under the curve and for the difference between areas. To assess the robustness of the cut-off, the entire cut-off selection and evaluation procedure was internally validated by bootstrap resampling, and optimism-corrected sensitivity, specificity, and accuracy were derived. The incremental value of the ratio beyond the Ranson score was explored by comparing the area under the curve of a combined logistic regression model (ratio plus Ranson score) with that of the Ranson score alone. To examine confounding by anemia, the association of the ratio with the target condition was adjusted for hemoglobin in logistic regression, and discrimination was additionally assessed within the non-anemic subgroup. A two-sided p-value below 0.05 was considered significant. Anemia was defined as hemoglobin below 13 g/dL in men and 12 g/dL in women. Bootstrap procedures used 3,000 resamples. Data were complete for the index test and the reference standard; CECT was unavailable in 13 patients, as described above.

Ethics

The study was approved by the Institutional Ethics Committee of Rajendra Institute of Medical Sciences, Ranchi (registration number: ECR/769/INST/JH/2015/RR-21) and was conducted in accordance with the 1964 Declaration of Helsinki and its later amendments [26]. Written informed consent was obtained from every participant.

## Results

Participants and baseline characteristics

A total of 142 patients were enrolled and analyzed; CECT was obtained in 129 (90.8%) (Figure 1). The mean age was 43.2 (SD: 12.6) years, with 79 (55.6%) patients aged 31-50 years, and 86 (60.6%) patients were men. Gallstones were the commonest etiology, including 64 (45.1%) patients, followed by alcohol, including 37 (26.1%) patients. By the Revised Atlanta Classification, 95 (66.9%) patients had mild disease, 25 (17.6%) moderately severe, and 22 (15.5%) severe; thus, 47 (33.1%) patients met the target condition. Baseline characteristics are summarized in Table 2. Sex and etiology did not differ between patients with and without the target condition, whereas shock at admission was more frequent in those meeting it (Table 3). Organ failure occurred in 32 (22.5%) patients, being respiratory in 18 (12.7%), cardiovascular in 7 (4.9%), and renal in 7 (4.9%), and was persistent in 22 (15.5%); pancreatic necrosis was present in 28 (19.7%). Moderately severe disease comprised transient organ failure in 10 (7.0%) patients and local or systemic complications without organ failure in 15 (10.6%), whereas all 22 (15.5%) patients with severe disease had persistent organ failure.

**Figure 1:**
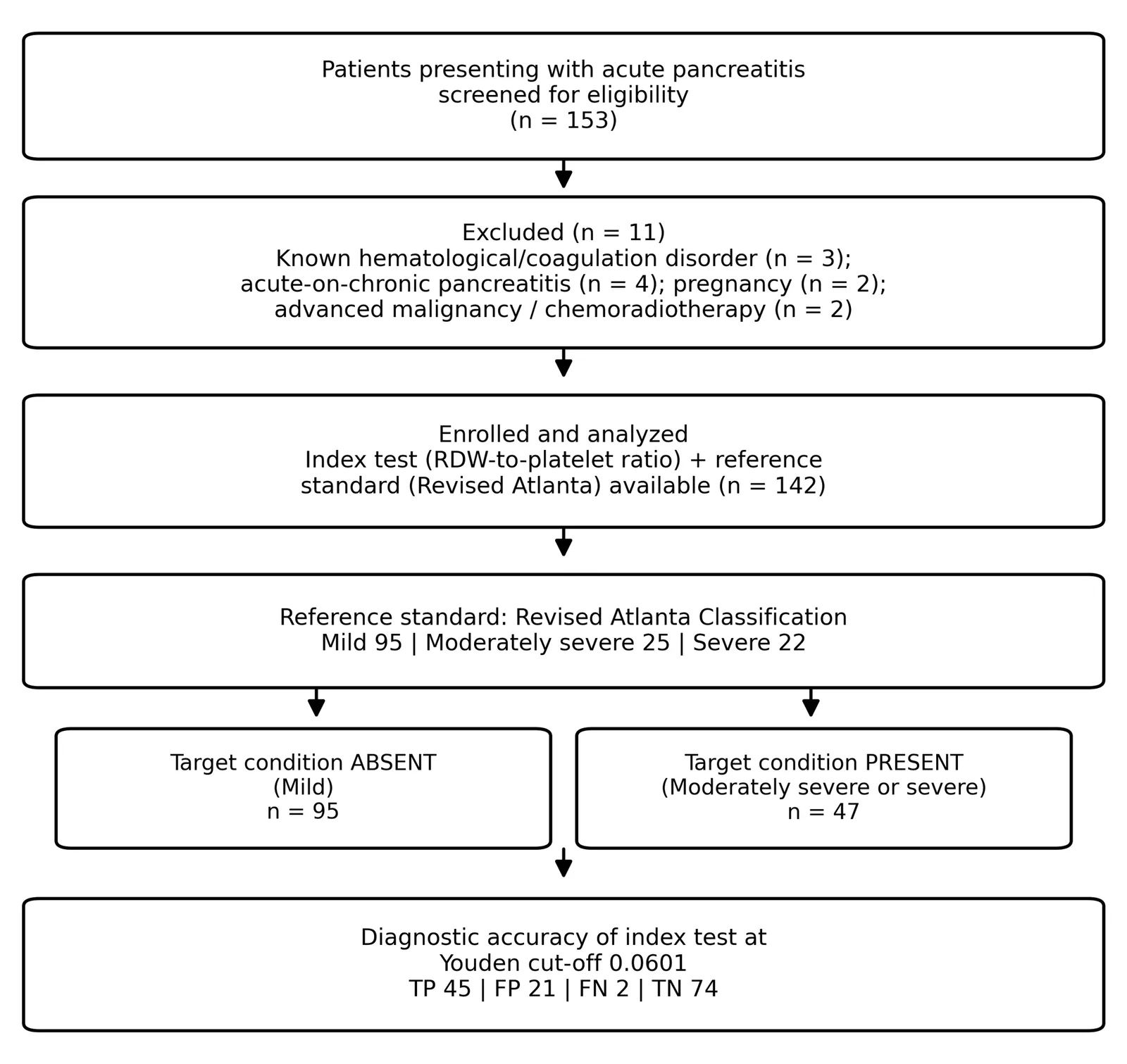
Study flow according to the STARD Of 153 patients screened, 11 were excluded and 142 were enrolled and analyzed. CECT: contrast-enhanced computed tomography, FN: false negative, FP: false positive, RDW: red cell distribution width, STARD: Standards for Reporting of Diagnostic Accuracy Studies, TN: true negative, TP: true positive

**Table 2:** Baseline demographic, clinical, and laboratory characteristics (n = 142) IQR: interquartile range, RDW: red cell distribution width, SD: standard deviation

Characteristic	Value
Age, years, mean (SD)	43.2 (12.6)
Men, number (%)	86 (60.6)
Etiology, number (%)	
Gallstone	64 (45.1)
Alcohol	37 (26.1)
Other	21 (14.8)
Idiopathic	20 (14.1)
Atlanta severity, number (%)	
Mild	95 (66.9)
Moderately severe	25 (17.6)
Severe	22 (15.5)
Shock at admission, number (%)	20 (14.1)
Red cell distribution width, %, median (IQR)	13.7 (13.0-14.8)
Platelet count, × 10⁹/L, mean (SD)	236.1 (72.0)
RDW-to-platelet ratio, median (IQR)	0.058 (0.047-0.080)
Ranson score, median (IQR)	2 (1-3)
Intensive care admission, number (%)	42 (29.6)
Organ failure, number (%)	32 (22.5)
In-hospital mortality, number (%)	13 (9.2)

**Table 3:** Categorical variables by target condition (moderately severe or severe versus mild acute pancreatitis) Percentages are column percentages within each target condition group. All comparisons used the Pearson chi-square test without continuity correction; the minimum expected cell count was 6.6, so no exact test was required. Shock at admission overlaps with the cardiovascular component of the Atlanta organ failure definition, so its association with the target condition is partly definitional and is reported for completeness rather than as an independent predictor. RDW: red cell distribution width

Variable	Category	Target condition present (n = 47), number (%)	Target condition absent (n = 95), number (%)	Test statistic, p	Cramér’s V
Sex	Male	27 (57.4)	59 (62.1)	χ² (1, N = 142) = 0.29, p = 0.59	0.04
	Female	20 (42.6)	36 (37.9)		
Etiology	Gallstone	22 (46.8)	42 (44.2)	χ² (3, N = 142) = 0.71, p = 0.87	0.07
	Alcohol	13 (27.7)	24 (25.3)		
	Other	7 (14.9)	14 (14.7)		
	Idiopathic	5 (10.6)	15 (15.8)		
Shock at admission	Yes	17 (36.2)	3 (3.2)	χ² (1, N = 142) = 28.32, p < 0.001	0.45
	No	30 (63.8)	92 (96.8)		
RDW-to-platelet ratio	≥0.0601	45 (95.7)	21 (22.1)	χ² (1, N = 142) = 68.54, p < 0.001	0.69
	<0.0601	2 (4.3)	74 (77.9)		

Association with severity and outcomes

The RDW-to-platelet ratio increased progressively across Atlanta categories, from a median of 0.050 in mild disease to 0.079 in moderately severe and 0.113 in severe disease (Kruskal-Wallis H = 73.5, p < 0.001), with all pairwise comparisons significant (all p < 0.001) (Figure 2, Table 4). The ratio was higher in patients classified as severe by the Ranson criteria than in those below the threshold (p < 0.001), in patients requiring intensive care than in those managed on the ward (p < 0.001), and in patients with organ failure than in those without (p < 0.001) (Table 4). It correlated moderately with the Ranson score (Spearman ρ = 0.51, p < 0.001) and with length of hospital stay (ρ = 0.57, p < 0.001).

**Figure 2:**
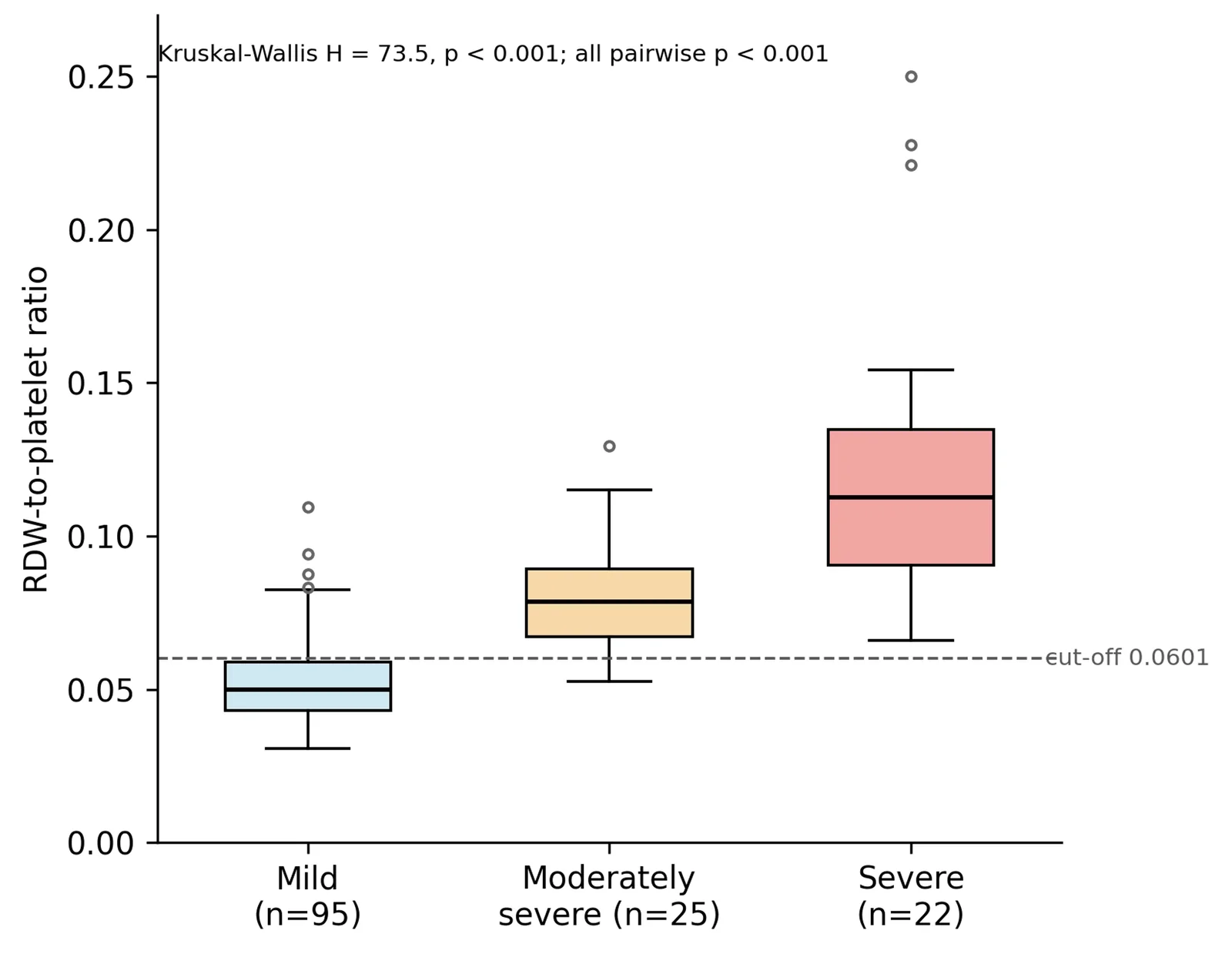
Distribution of the RDW-to-platelet ratio across Revised Atlanta severity categories The dashed line marks the diagnostic cut-off of 0.0601. RDW: red cell distribution width

**Table 4:** RDW-to-platelet ratio by severity category and clinical outcome (n = 142) H: Kruskal-Wallis statistic, with Dwass-Steel-Critchlow-Fligner pairwise comparisons (all p < 0.001) U: Mann-Whitney statistic Correlation of the ratio with the Ranson score (Spearman ρ = 0.51) and with length of hospital stay (ρ = 0.57), both p < 0.001 IQR: interquartile range; RDW: red cell distribution width

Comparison	Group (number)	RDW-to-platelet ratio, median (IQR)	Statistic, p
Atlanta severity	Mild (95)	0.050 (0.043-0.059)	H(2) = 73.5, p < 0.001
	Moderately severe (25)	0.079 (0.067-0.089)	
	Severe (22)	0.113 (0.091-0.135)	
Ranson category	<3 (101)	0.052 (0.044-0.061)	U = 385.5, p < 0.001
	≥3 (41)	0.090 (0.070-0.114)	
Intensive care	No (100)	0.052 (0.044-0.064)	U = 592.5, p < 0.001
	Yes (42)	0.089 (0.067-0.115)	
Organ failure	No (110)	0.053 (0.044-0.065)	U = 250.5, p < 0.001
	Yes (32)	0.097 (0.082-0.127)	

Diagnostic accuracy

For identifying moderately severe or severe disease, the admission ratio achieved an area under the curve of 0.931 (95% confidence interval: 0.888-0.966) (Figure 3, Table 5). At the Youden-derived cut-off of 0.0601, sensitivity was 95.7% (45/47, 85.8-98.8), specificity 77.9% (74/95, 68.6-85.1), positive predictive value 68.2% (45/66, 56.2-78.2), negative predictive value 97.4% (74/76, 90.9-99.3), and overall accuracy 83.8% (119/142, 76.9-89.0). The Ranson score had a higher area under the curve (0.979, 0.960-0.994); the difference of 0.048 (0.008-0.096) was statistically significant (bootstrap p = 0.018), indicating that the ratio, although an excellent discriminator, did not match the multiparameter 48-hour score. Dichotomized at this cut-off, the ratio was strongly associated with the target condition (χ²(1, N = 142) = 68.54, p < 0.001, Cramér’s V = 0.69). The ratio discriminated better than either component alone (area under the curve: 0.857, 95% confidence interval: 0.771-0.931 for RDW and 0.869, 0.808-0.922 for platelet count).

**Figure 3:**
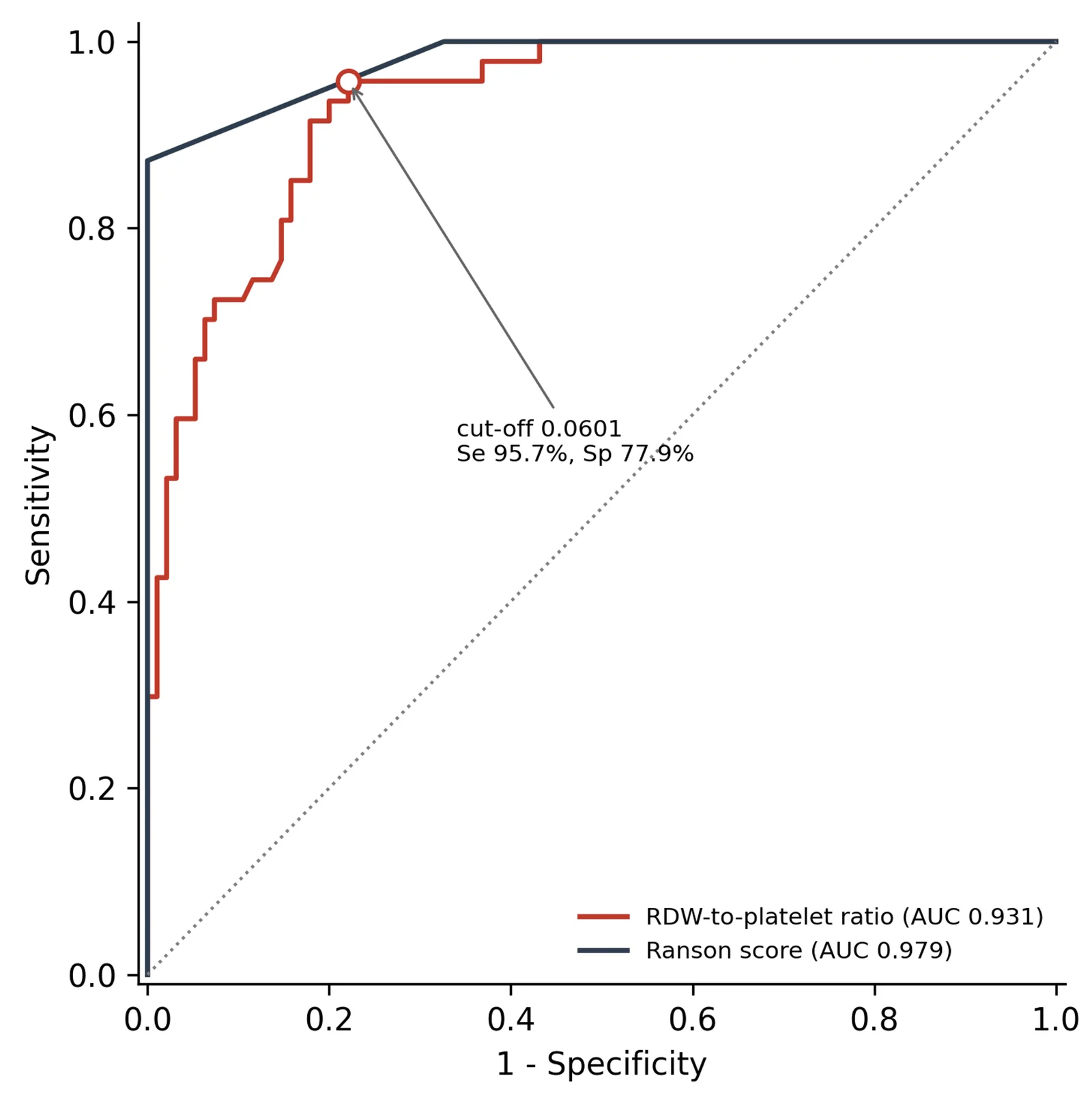
Receiver operating characteristic curves of the RDW-to-platelet ratio and the Ranson score for identifying moderately severe or severe acute pancreatitis AUC: area under the curve, RDW: red cell distribution width

**Table 5:** Diagnostic performance for identifying moderately severe or severe acute pancreatitis (n = 142) Difference in area under the curve (Ranson minus ratio): 0.048 (95% CI: 0.008-0.096), bootstrap p = 0.018 CI: confidence interval, RDW: red cell distribution width

Measure	RDW-to-platelet ratio	Ranson score
Area under the curve (95% CI)	0.931 (0.888-0.966)	0.979 (0.960-0.994)
Optimal cut-off (Youden)	0.0601	-
Sensitivity, n/N (%, 95% CI)	45/47 (95.7, 85.8-98.8)	-
Specificity, n/N (%, 95% CI)	74/95 (77.9, 68.6-85.1)	-
Positive predictive value, n/N (%, 95% CI)	45/66 (68.2, 56.2-78.2)	-
Negative predictive value, n/N (%, 95% CI)	74/76 (97.4, 90.9-99.3)	-
Accuracy, n/N (%, 95% CI)	119/142 (83.8, 76.9-89.0)	-

Sensitivity and internal validation analyses

In internal validation by bootstrap resampling, the cut-off performed consistently, with an optimism-corrected sensitivity of 93.2%, specificity of 77.0%, and accuracy of 82.4%, close to the apparent estimates. When the admission ratio was added to the Ranson score in a combined model, discrimination improved beyond the Ranson score alone (combined area under the curve: 0.998; increment: 0.019, 95% confidence interval: 0.005-0.036), indicating that the ratio carried information additional to the score. The association of the ratio with the target condition persisted after adjustment for hemoglobin (adjusted odds ratio per 1 SD: 15.5, 95% confidence interval: 9.2-29.5), and discrimination remained high in both non-anemic (n = 64) and anemic patients (n = 78) (area under the curve: 0.899 and 0.954, respectively), indicating that performance was not explained by anemia. When the target condition was restricted to severe disease alone, the area under the curve was 0.947 at a higher optimal cut-off of 0.0893 with sensitivity of 81.8% (18/22) and specificity of 92.5% (111/120).

Temporal change

Between admission and 48 hours, the RDW rose and the platelet count fell, with a corresponding significant increase in the ratio (Wilcoxon p < 0.001), although the median change was modest (Table 6). These directional changes are consistent with an evolving inflammatory process over the early disease course.

**Table 6:** Change in hematological parameters from admission to 48 hours (n = 142) p-values from the Wilcoxon signed-rank test RDW: red cell distribution width

Parameter	Admission, median	48 hours, median	P
Red cell distribution width, %	13.7	14.0	<0.001
Platelet count, × 10⁹/L	235	229	<0.001
RDW-to-platelet ratio	0.058	0.060	<0.001

## Discussion

In this prospective cohort, the admission RDW-to-platelet ratio rose stepwise across Revised Atlanta severity categories and was consistently higher in patients who required intensive care or developed organ failure. It discriminated moderately severe or severe disease with an area under the curve of 0.931 and, at a cut-off of 0.0601, combined high sensitivity with a negative predictive value approaching 97% (74/76). The ratio is derived from two components of the routine complete blood count, requires no additional cost, and is available within hours of admission, making it well suited to early triage.

These findings are biologically coherent. RDW increases with systemic inflammation, oxidative stress, and impaired erythropoiesis, all of which accompany severe pancreatitis, and correlates with circulating inflammatory markers [11-13]. The platelet count may fall through consumption and microvascular sequestration during intense inflammation. Combining the two into a single ratio captures both directions of change and may therefore track the systemic inflammatory burden more sensitively than either component alone. The stepwise rise across severity strata and the strong separation by organ failure status observed here support this rationale.

The results are consistent with the direction of earlier reports. Preliminary studies described prognostic value for the ratio in acute pancreatitis [16,17], and prospective and cross-sectional cohorts, several published recently, have since related it to severity and outcomes [27-29], including acute biliary pancreatitis [30]. Against this growing body of work, the present study contributes a prospective cohort from the Indian subcontinent evaluated against the three-tier Revised Atlanta reference standard, with the platelet denominator units stated explicitly and the cut-off subjected to internal validation, features that are inconsistently reported elsewhere. Related RDW ratios using serum calcium or albumin have shown comparable discrimination [19-21], and the parent marker predicts persistent organ failure and death in acute pancreatitis [14,15]. A central caveat, however, is that reported cut-off values differ by orders of magnitude between studies because the platelet denominator is expressed inconsistently, as an absolute count in some and as × 10⁹/L in others. Absolute cut-offs are therefore not transferable, and comparison across studies should rest on discrimination metrics such as the area under the curve rather than on the numerical threshold. This study states the denominator units explicitly for that reason.

The comparison with the Ranson score requires a measured interpretation. Although both markers discriminated severity well, the Ranson score achieved a significantly higher area under the curve. This is unsurprising, as the Ranson score aggregates multiple physiological and biochemical variables collected over 48 hours, whereas the ratio derives from a single admission sample. The value of the ratio therefore lies not in replacing composite scores but in providing rapid, early information during the initial hours, before 48-hour data or definitive imaging are available. Its high negative predictive value suggests potential as an early rule-out marker: a low admission ratio identified patients unlikely to have clinically significant disease in this cohort. Whether the ratio can inform level-of-care decisions was not evaluated here and remains a hypothesis for prospective study; it should be interpreted alongside clinical status and established scores rather than in isolation.

Notably, adding the ratio to the Ranson score improved discrimination beyond the score alone, suggesting that the two capture partly complementary information and that the ratio may contribute both as an early stand-alone signal and as a component of composite assessment. These findings nonetheless derive from an accuracy study and do not themselves demonstrate that acting on the ratio changes management or outcomes; its role is best regarded as an adjunct to clinical judgment rather than as a trigger for specific interventions. The high negative predictive value also reflects the 33% (47/142) prevalence of clinically significant severity in this cohort and will vary with case mix.

This study has strengths. Data were captured prospectively at predefined time points, reducing recall bias; severity was assigned with a standardized classification and benchmarked against a validated score and clinically meaningful outcomes; the index test is inexpensive and reproducible; and diagnostic estimates are reported with confidence intervals.

Several limitations temper the findings. As a single-center study, the case mix and referral pattern may limit generalizability, and external validation is required. Although the cut-off was internally validated with only modest optimism, it was derived and evaluated in a single cohort and still requires confirmation in independent data before adoption. Not all patients underwent CECT; severity in those without imaging was assigned from clinical, biochemical, and organ failure criteria, which may introduce misclassification for local complications. RDW and platelet count can be influenced by conditions unrelated to pancreatitis; although the ratio remained associated with severity after adjustment for hemoglobin, other factors such as chronic inflammation, infection, and liver disease were not fully accounted for. Severe events, particularly mortality, occurred in relatively few patients, so the confidence intervals for outcome-specific estimates are correspondingly wide. Finally, the incremental value of the ratio beyond existing scores was explored but not prospectively validated, and combined models and serial measurement strategies require confirmation. The combined model was derived and evaluated in the same cohort, so its near-perfect discrimination is likely optimistic. The ratio was not compared with admission-based composite scores such as BISAP or APACHE II, which were not recorded. A high negative predictive value does not itself establish that the ratio can safely guide ward placement or exclude subsequent deterioration.

Future work should validate the cut-off in multicenter prospective cohorts, evaluate whether the ratio adds incremental value to established scores within combined models, and test whether serial change improves prediction of deterioration. Standardized reporting of the platelet denominator across studies would allow meaningful comparison of thresholds.

## Conclusions

The admission RDW-to-platelet ratio is a simple, inexpensive, and rapidly available marker that rises with the severity of acute pancreatitis and is associated with intensive care admission, organ failure, and prolonged hospital stay. Its high negative predictive value suggests potential as an adjunctive early marker to help exclude clinically significant severity, complementing rather than replacing established scoring systems in the initial assessment of acute pancreatitis. External, multicenter validation with standardized reporting of the platelet denominator is required before routine clinical use.
